# Supplementary material for: Identification of Neuronal Polarity by Node-Based Machine Learning
Source: Neuroinformatics. 2021 Mar 5;19(4):669–84. doi: 10.1007/s12021-021-09513-y (PMC8566381; doi:10.1007/s12021-021-09513-y)
Supplement: Supplementary file 1 — (PDF 2.31 mb) [file 12021_2021_9513_MOESM1_ESM.pdf]

Appendix

A. Generation of a Reduced Tree from a Level Tree

The level tree defined in this paper (see *Standardized Representation: Level Trees and Reduced Trees* and Fig. 3) has contained enough neuronal information for the determination of the nodal polarity. However, there are in general so many levels and nodes are involved that certain important features, like "clusters", cannot be defined easily from a computational point of view. In order to have a better definition of Local Features, which are more related to the geometric nature of the domain a node belongs to, in this paper we use a systematic method to obtain a "cluster" by trimming less important branches and then keeping the main trunk of a neuron. Those nodes left in such reduced trees on the trunks are then defined to be "the heads of clusters" (see for example, the node *i* in Fig. S2(b) below). We then use their spatial information to define "clusters" and calculate Local Features.

The way we trim less important branches is described as following steps: First, we calculate the number distribution of the whole "leaves", which are the branches to connect each terminal (see Fig. S1(a)) and their upper level nodes for a given neuron, as a function of their path lengths. From this distribution, we could determine the characteristic length, which has the largest number of "leaves" than other lengths. Second, we trim those "leaves" if their length is smaller than the characteristic length mentioned above. As a result, there will be new leaves coming out for new "terminals" after such trimming in the first round. Third, we repeat the calculation of length distribution again and trim those "leaves" of relatively shorter path lengths. Finally, such a trimming process is terminated when no more "leaves" of path lengths shorter than the characteristic length in the distribution, or when the total number of levels in the reduced tree reaches five (which is a convention we choose according to experience). In the inset of Figs. S1(a) and S1(b) we show the skeleton structure of a complex neuron before and after such reduction for comparison. The difference between these two figures give us the information of "clusters". The level tree and reduced tree representation are then obtained from these two skeleton structures respectively.

It is easy to see that this is an efficient and systematic way to determine "clusters" from the level tree, while there are certainly also other methods to define clusters. However, we have to emphasize that the identification of nodal polarity should not be sensitive to the details of these definitions, because we use these "clusters" to calculate morphological features, such as aspect ratio and curvature (see Appendix B), instead of using their detailed information directly. The identification of nodal polarity is determined not only by using Local Features and Soma Features, but also by implementing spatial correlation of polarities between neighboring nodes.

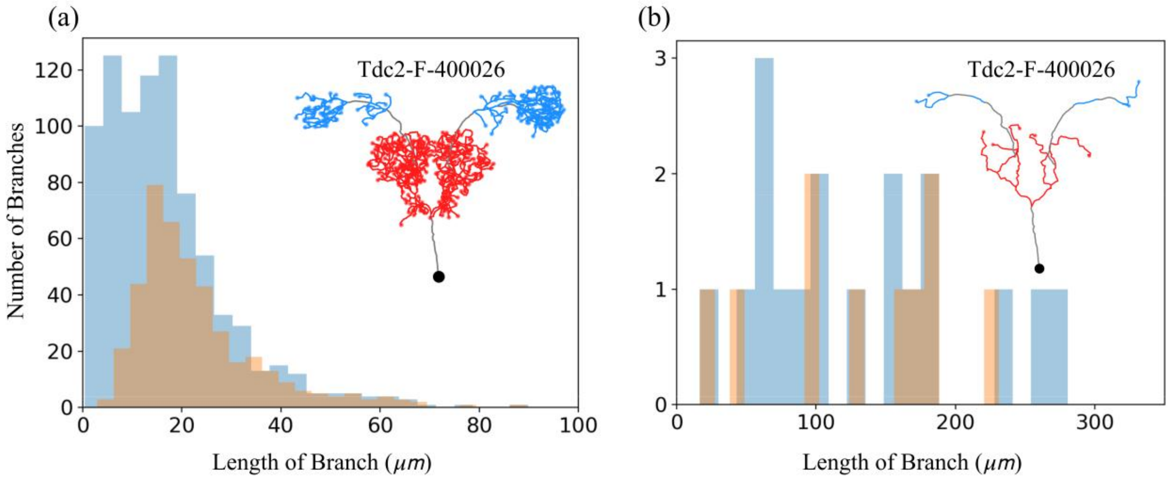

Fig. S1 (a) shows the distribution of path length of all branches (blue) and all "leaves" (orange) of a given neuron. The threshold of trimming length is determined by the maximum value of the distribution for the "leaves". In this case, it is around 15 μm in the beginning. (b) shows the final distribution of all branches and "leaves" after repeating the trimming process. Since shorter and outer branches (leaves) are mostly trimmed, the final branches (after combining two or more branches) also become longer. The insets in (a) and (b) show the skeleton structure before and after such a process. The level tree and reduced tree representations (shown in Fig. 3) are obtained from these two skeleton structures respectively.

B. Calculation of Local Features: Curvature and Aspect Ratio

In this paper, we define two types of features for the polarity identification, Soma Features and Local Features respectively. It is easier to describe them in a skeleton diagram of neurons as shown in Figs. S2: For a given node, which is labeled as i here, Soma Features contain four values: (1) path length (along the neuron branch) to soma, l_{si}, see the thick line along S-p-i, (2) normalized path length to soma, $nl_{si} = l_{si}/L_s$, where L_s is the largest path length to soma for a given neuron. (3) direct distance to soma, d_{si}, see the dashed straight line, S-i, and (4) normalized distance to soma, $nd_{si} = d_{si}/D_s$, where D_s is the largest distance to soma for a given neuron. Note that we include both original path length/distance to soma and their normalized values because the former is to catch the possible size effects between different neurons, while the latter is for the comparison between nodes of the same neuron. These four features are defined to be Soma Features in this paper, because they are all related to the spatial information between nodes to soma.

Here we have to emphasize that Soma Features defined above contain not only the distance information for a node to soma, but also certain global shape information. The reason is that both path length to soma and direct distance to soma are included, and their ratio could imply how the neuronal truck and branches are curved in space between the given node and soma. In other words, our Soma Features provides more information than just the path length to soma. If using normalized path length to soma as the only feature for comparison, the accuracy of polarity prediction drops to 91.3% (by XGB), which is much lower than the accuracy (95.5%) obtained by using Soma Features defined above.

For Local Features, in this paper we include the following five values for a given node (see Fig. S2(b)): (1) path length to its parent node (in the upper level if in the level tree diagram), l_{pi}, see the thick line p-i, (2) normalized path length to its parent node, i.e. $nl_{pi} = l_{pi}/L_s$, (3) the curvature of the associated cluster, c_i, (4) the aspect ratio of the associated cluster, ar_i, and (5) the ratio of length, $rl_i = Min(l_{ij}, l_{ik})/(l_{ij} + l_{ik})$, which is to measure the relative differences between the lengths to its two children nodes. In Fig. S2(c), we show how to calculate the curvature and aspect ratio of a given "cluster", which is obtained from the definition of our reduced trees (see Appendix A). Note that all the Local Features are related only to the path length or shape structure of a given node with its neighboring nodes, and contains *no* information about its relationship to soma at all.

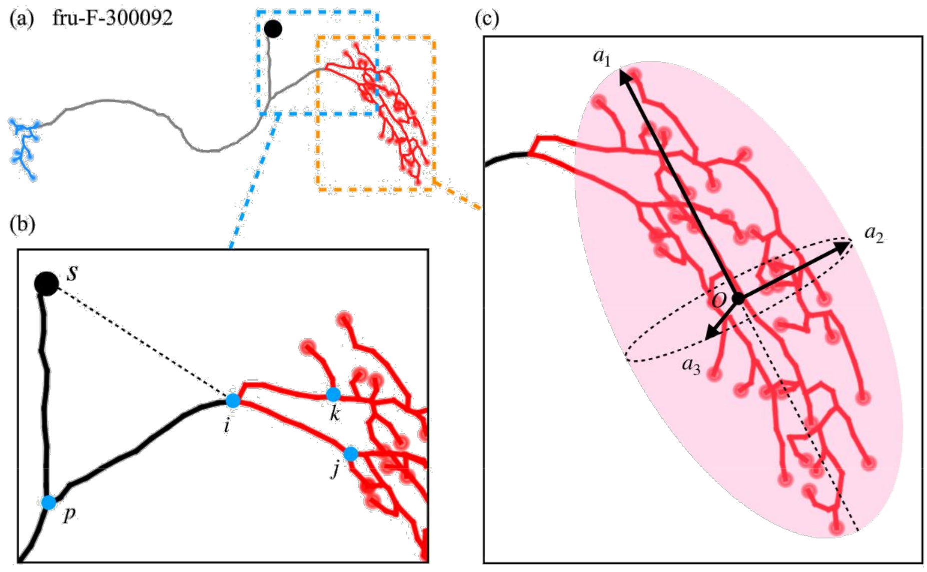

Fig. S2 (a) shows a typical skeleton structure of a neuron in our dataset. In (b), we zoom in the neighboring skeleton structure of a given node, which is labeled by the index i. The path length to soma, direct distance to soma and the path length to other lower level nodes are denoted (see the text for details). In (c), we show the local morphological features of a given node inside the cluster. The curvature feature is defined by the ratio of total neuronal path length inside the cluster to the size of the cluster. This value indicates how much the neuronal branches are packed inside the cluster. The aspect ratio is to calculate the ratio of the longest length and the shortest length of a 3D elliptic surface, obtained according to the calculation of moment of inertia for this cluster. The details of calculation are described in the text.

In order to incorporate local morphological information for each node of a neuronal skeleton, two local features, curvature and aspect ratio, are defined for the "cluster", where a given node belongs to. The definition of "cluster" in this paper has been described in Appendix A and Fig. S1 above. To calculate the aspect ratio of such "cluster", we assume each node (including terminals) inside the cluster is a point of the same "mass", and therefore the moment of inertia with respect to their center of mass is given by the following two-rank tensor:

$$I = \begin{bmatrix} I_{xx} & I_{xy} & I_{xz} \\ I_{yx} & I_{yy} & I_{yz} \\ I_{zx} & I_{zy} & I_{zz} \end{bmatrix}$$

where each element can be calculated from

$$I_{xx} = \sum_{k \in cluster} (y_k^2 + z_k^2), I_{yy} = \sum_{k \in cluster} (x_k^2 + z_k^2), I_{zz} = \sum_{k \in cluster} (x_k^2 + y_k^2)$$

$$I_{xy} = I_{yx} = - \sum_{k \in cluster} x_k y_k,\ I_{xz} = I_{zx} = - \sum_{k \in cluster} x_k z_k,\ I_{yz} = I_{zy} = - \sum_{k \in cluster} y_k z_k$$

Here x_k, y_k, and z_k are the position coordinates for each node (with a dummy index, k) relative to their center of mass point, O, which can be calculated easily (see Fig. S2(c)).

After diagonalization of this moment of inertia tensor, the obtained eigenvectors are orthogonal to each other and can be denoted to be $a_{1,2,3}$ as the three principal axes. Therefore, the aspect ratio of the cluster can be defined as $r \equiv Max[a_i/a_j]$ (see Fig. S2(c) as an example). After finding the length scale of the associated cluster, we can further define "curvature" of such cluster as, $c \equiv (total\ pathlength\ in\ the\ cluster)/(a_1 a_2 a_3)^{1/3}$, which indicates how tightly the nerve branches are packed inside such a cluster.

C. Machine Learning Algorithms

In this paper, we have used two machine learning algorithms, XGB and DNN. XGB uses gradient boosting methods based on a rule-based algorithm in order to optimize the accuracy in many-different tasks[1] [2]. It belongs to so-called transparent models, because the algorithm could automatically evaluate the weighting of each feature and decide the best arrangement for each decision tree (See Fig. S3(a)). As a result, XGB could also provide feature ranking to evaluate the importance of these input features. In our NPIN, the XGB algorithm is designed with default hyper parameters from XGBoost Python Package: the learning rate is 0.1, maximum depth of each tree is 3, and the number of trees is 100.

DNN was an algorithm originally designed to mimic the architecture of the human neural networks, but became one of the most general and powerful algorithms in the field of Artificial Intelligence[3] [4] [5]. Its common structure is composed of one input layer and one output layer, in between are multiple hidden layers (see Fig. S3(b)). In each layer, there are a number of interconnected nodes, or called "artificial neurons", which receive inputs from "artificial neurons" in the previous layer and supply outputs to others in the next layer. Each node performs a weighted sum computation on the values it receives from the input and then generates an output after a nonlinear transformation

[1] Bekkerman, R. (2015). The Present and the Future of the KDD Cup Competition: an Outsider's Perspective. https://www.linkedin.com/pulse/present-future-kdd-cup-competition-outsiders-ron-bekkerman/. Accessed 29 April 2020

[2] Chen, T., & Guestrin, C. (2016). XGBoost: A Scalable Tree Boosting System. In *Proceedings of the 22nd ACM SIGKDD International Conference on Knowledge Discovery and Data Mining*(pp. 785–794). San Francisco, California, USA: Association for Computing Machinery. https://doi.org/10.1145/2939672.2939785

[3] Deng, L., & Yu, D. (2014). Deep Learning: Methods and Applications. *Foundations and Trends® in Signal Processing*,*7*(3–4), 197–387. https://doi.org/10.1561/2000000039

[4] LeCun, Y., Bengio, Y., & Hinton, G. (2015). Deep learning. *Nature*,*521*(7553), 436–444. https://doi.org/10.1038/nature14539

[5] Schmidhuber, J. (2015). Deep learning in neural networks: An overview. *Neural Networks*,*61*, 85–117. https://doi.org/10.1016/j.neunet.2014.09.003

function on the summation. In our NPIN, the DNN algorithm has 9 features in the input layer (four Soma Features and five Local Features), propagated directly to the two fully connected hidden layers. Both of them contain ten artificial neurons and apply sigmoid function as activation functions. The output layer has two artificial neurons representing the probability of being axon or dendrite, using Focal Loss function[6] and Adam optimizer[7] for the training processes.

Therefore, we can see that XGB and DNN are complementary algorithms in many different perspectives: First of all, XGB is a transparent model, which could provide certain results for feature ranking, while DNN is known not explainable due to its highly non-linear function coupling. Besides, it is known that XGB does not need much data (as well as computational time) for the training process, while DNN usually needs a large amount of data and computational power. On the other hand, when the amount of training data is sufficiently large, DNN in general provides higher precision than transparent models, especially when the features are highly correlated to each other.

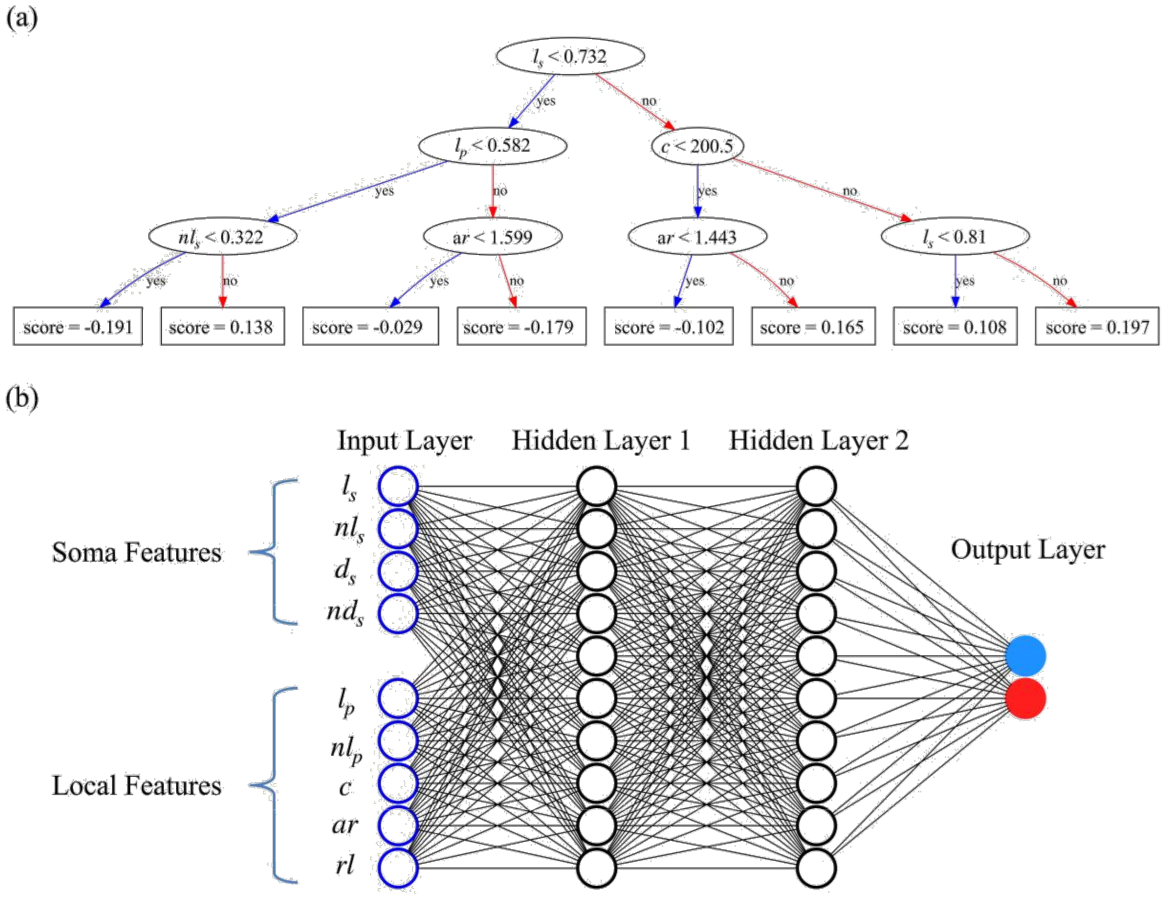

Fig. S3 (a) shows a typical decision tree diagram, obtained by XGB with feature ranking. Each oval represents a criterion and the scores from all the decision trees are used for calculating the probability to be axon (higher score) or dendrite (lower scores) (Chen and Guestrin 2016). (b) shows the layer structure of our DNN model, where we use fully connecting networks between artificial neurons in the neighboring layers.

D. Including Spatial Nodal Correlation through Polarity Relabeling

In the NPIN model, the accuracy of polarity classification can be further improved by imposing the spatial correlation between nodal polarities. This is because, except for some local neurons which are not included in our analysis, axonal or dendritic terminals in a neuron appear in clusters. However, in our XGB or DNN algorithms, the polarity of each node is independently classified without knowing polarities of neighboring nodes. The spatial correlation is then imposed by relabeling the polarities of nodes, which have low probability scores from the classification process, and the polarity of a relabeled node is determined by its neighboring nodes that have higher probability scores.

---

[6] Lin, T.-Y., Goyal, P., Girshick, R., He, K., & Dollár, P. (2018). Focal Loss for Dense Object Detection. *arXiv:1708.02002 [cs]*.http://arxiv.org/abs/1708.02002. Accessed 29 April 2020
[7] Kingma, D. P., & Ba, J. (2017). Adam: A Method for Stochastic Optimization. *arXiv:1412.6980 [cs]*. http://arxiv.org/abs/1412.6980. Accessed 29 April 2020

The basic relabeling procedures are following: First we relabel the polarity of all the nodes according to the nodal polarity in the lower level, see Fig. S4. In other words, although we could predict all the nodal polarity as well as the terminal polarity by XGB or DNN, the terminals of high probabilities to be axons or dendrites still have higher priority to determine the polarity of nodes in the upper levels. On the other hand, those terminals predicted to have lower probabilities to be either axons or dendrites will then be determined from nodes in the upper levels and its neighbors in the second stage. Finally, we define dividing nodes in the same way as we described for the data preparation (see Fig. 3(c)). This stage will not rewrite any polarity except to identify dividing nodes in order to make the level tree representation consistent with the original definition.

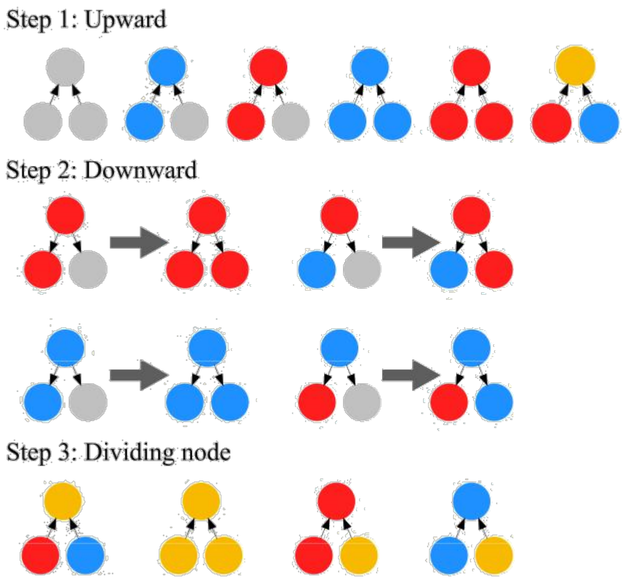

Fig. S4 shows the three steps to relabel nodal polarity by including the spatial correlation between neighboring nodes. Grey nodes are nodes with low probability scores from the polarity classification, green nodes are classified axons, red nodes are classified dendrites and purple nodes are the dividing nodes. In Step 1, nodal polarity is determined by the polarity of nodes in the lower level, taking into account the existence of nodes with probabilities lower than the threshold (filled grey circles). In Step 2, grey circles are relabeled according to the results of its parent nodes in the upper level. In Step 3, we complete the relabeling process by identifying dividing nodes. The final results are consistent with the original definition of nodal polarity shown in Fig. 3(c), but we use the polarities of terminal nodes only to get the ground truth.

In order to determine the criteria for a node to enter the relabeling process, in Fig. S5(a) we plot the distribution of nodes based on the probability being an axon from the XGB classifier. Since it is a binary classification, a higher probability to be an axon must indicate a lower probability to be a dendrite, and vice versa. We can see that most nodes are classified to be either axon or dendrite with high probabilities (as shown by peaks on the two sides), but there are still a small portion of nodes with low probabilities. In Fig. S5(b1). We show the level tree of a given neuron with preliminary results given by XGB. One could see that although most nodal polarities are correctly identified, some are incorrectly identified. In Fig. S5(b2) we show the same level tree by removing the polarity labels of nodes which have probabilities being axons or dendrites below 0.75. In Fig. S5(b3), we show the final results of the level tree after relabeling (see Fig. S4): all nodes now are correctly classified after imposing spatial correlation through the relabeling process.

In Figs. S5(c) and S5(d) we show the comparison of the precision/recall table before and after such relabeling. It shows that the spatial correlation may significantly enhance the recall of axons by XGB, while it does not enhance that much for DNN. Since the spatial correlation is also strongly related to the distribution of neighboring nodes, the above result reflects the fact that, in a rule-based algorithm like XGB, morphologic features (curvature and aspect ratio) are relatively less addressed. However, spatial correlation can be learned to some degree through these morphologic features in DNN, making such post-relabeling less effective.

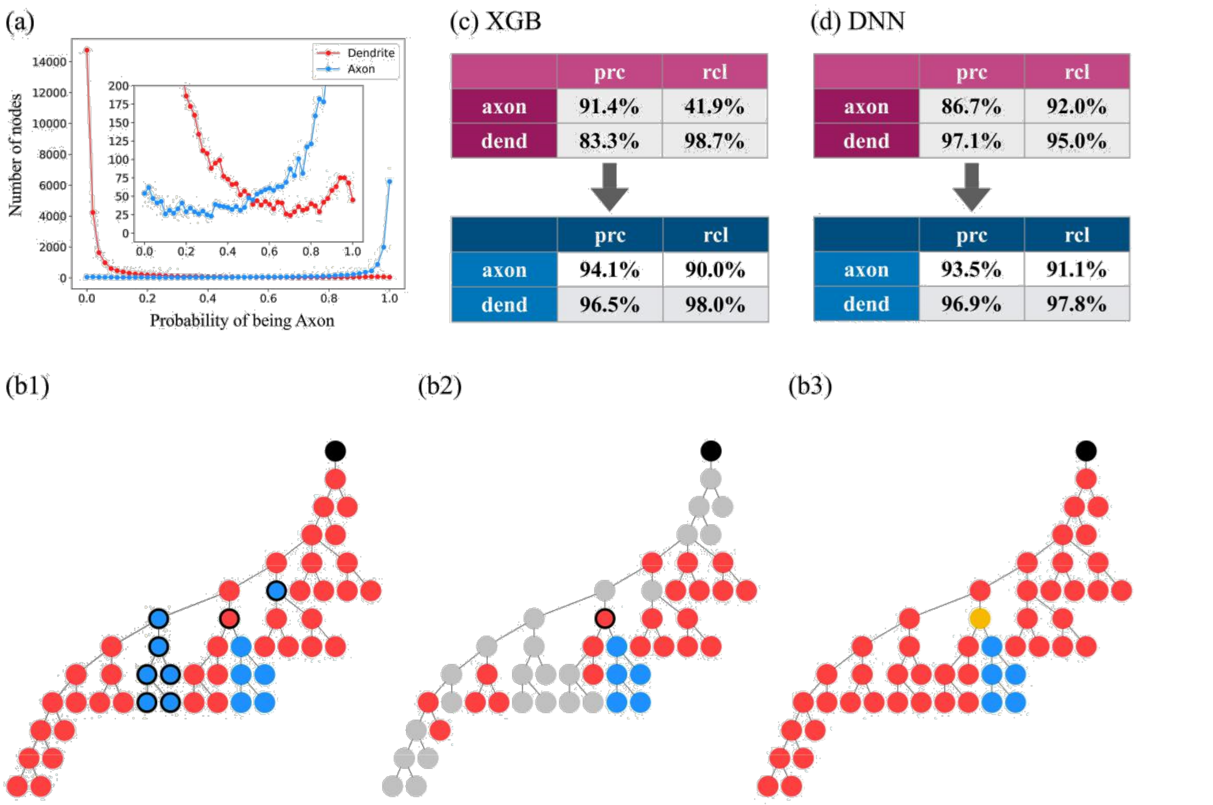

(c) XGB

	prc	rcl
axon	91.4%	41.9%
dend	83.3%	98.7%

	prc	rcl
axon	94.1%	90.0%
dend	96.5%	98.0%

(d) DNN

	prc	rcl
axon	86.7%	92.0%
dend	97.1%	95.0%

	prc	rcl
axon	93.5%	91.1%
dend	96.9%	97.8%

Fig. S5 (a) shows the distributions of the probability being an axon as classified by XGB. The distributions are plotted separately for nodes that are actually axons (green) or dendrites (red) for all 213 neurons in our dataset. Note that this is a binary classification, so the sum of the probability to be dendrite and the probability to be axon has to be equal to one. (b1) is the level trees of the preliminary result given by XGB, where some nodes are incorrectly identified, as indicated by thick black circles. (b2) is the same level tree with removed polarity labels for some nodes (grey), if their probabilities be an axon and a dendrite are both below a threshold (=0.75). (b3) shows the final results after relabeling. All the axons and dendrites are correctly classified after imposing the spatial correlation of nodal polarity. (c) and (d) are the precision/recall tables provided by XGB and DNN, respectively for Model I (using both Soma Features and Local Features), applied on the whole dataset. The upper tables are the results before the relabeling process and the lower ones are after.

E. Neuron Dataset for NPIN and Their Polarity Identification Results

Here we present the results of polarity identification by NPIN for all neurons in our dataset. It is obtained by randomly selecting 150 neurons (100 for training, 25 for validation, and 50 for testing) out of the 213 neurons in the dataset for each training/test process, and we repeat the same calculation for 20 rounds. As a result, each neuron can be tested (by different models trained by other neurons) for 4 or 5 times on average and the terminal polarities are identified by averaging their probabilities before final relabeling.

Tabel S1. Polarity Identification of Simple Neurons in the Dataset

Neuron ID	Brain Region		No. of Terminals		Precision		Recall		Accuracy
	Axon	Dendrite	Axon	Dendrite	Axon	Dendrite	Axon	Dendrite	
5HT1A-F-100004	LH	AL	18	64	1.00	1.00	1.00	1.00	1.00
5HT1A-F-100032	LH	AL	23	132	1.00	1.00	1.00	1.00	1.00
5HT1A-F-600006	LH	AL	11	29	1.00	1.00	1.00	1.00	1.00
Cha-F-100132	AOTU	MED	2	55	1.00	1.00	1.00	1.00	1.00
Cha-F-100165	AOTU	MED	3	28	1.00	1.00	1.00	1.00	1.00
Cha-F-100352	AOTU	MED	2	20	1.00	1.00	1.00	1.00	1.00
Cha-F-100414	AOTU	MED	2	38	1.00	1.00	1.00	1.00	1.00
Cha-F-300281	AOTU	MED	6	104	1.00	1.00	1.00	1.00	1.00
Cha-F-500233	AOTU	MED	4	59	1.00	1.00	1.00	1.00	1.00
Cha-F-600158	AOTU	MED	2	36	1.00	1.00	1.00	1.00	1.00
Cha-F-600239	AOTU	MED	3	48	1.00	1.00	1.00	1.00	1.00
Cha-F-600263	AOTU	MED	4	63	1.00	1.00	1.00	1.00	1.00
Cha-F-700223	AOTU	MED	1	43	1.00	1.00	1.00	1.00	1.00
Cha-F-800020	AOTU	MED	2	69	1.00	1.00	1.00	1.00	1.00
Cha-F-800097	AOTU	LOB	9	37	1.00	1.00	1.00	1.00	1.00
fru-F-000012	VMP	MED	5	25	1.00	1.00	1.00	1.00	1.00
fru-F-000230	VMP	MED	4	49	1.00	1.00	1.00	1.00	1.00
fru-F-100050	VMP	MED	10	69	1.00	1.00	1.00	1.00	1.00
fru-F-100096	VMP	MED	6	39	1.00	1.00	1.00	1.00	1.00
fru-F-200085	VMP	MED	9	54	1.00	1.00	1.00	1.00	1.00
fru-F-200101	VMP	MED	8	71	1.00	1.00	1.00	1.00	1.00
fru-F-200153	VMP	MED	10	105	1.00	1.00	1.00	1.00	1.00
fru-F-300092	VMP	MED	8	36	1.00	1.00	1.00	1.00	1.00
fru-F-300111	VMP	MED	9	78	1.00	1.00	1.00	1.00	1.00
fru-F-300120	VMP	MED	9	52	1.00	1.00	1.00	1.00	1.00
fru-F-300130	VMP	MED	4	79	1.00	1.00	1.00	1.00	1.00
fru-F-400057	VMP	MED	5	21	.833	1.00	1.00	.952	.962
fru-F-400058	AOTU	LOB	7	14	1.00	1.00	1.00	1.00	1.00
fru-F-400209	AOTU	LOB	10	22	1.00	1.00	1.00	1.00	1.00
fru-F-400341	VMP	MED	6	44	1.00	1.00	1.00	1.00	1.00
fru-F-400366	VMP	MED	5	93	1.00	1.00	1.00	1.00	1.00
fru-F-500119	AOTU	LOB	6	8	1.00	1.00	1.00	1.00	1.00
fru-F-500257	PB	CVLP,DMP	12	83	.857	.928	.500	.987	.922
fru-F-500486	AOTU	LOB	2	12	1.00	1.00	1.00	1.00	1.00
fru-F-500578	VMP	MED	7	55	1.00	1.00	1.00	1.00	1.00

fru-F-600005	VMP	MED	8	31	1.00	1.00	1.00	1.00	1.00
fru-F-700013	VMP	MED	8	28	1.00	1.00	1.00	1.00	1.00
fru-F-700059	VMP	MED	10	39	.909	1.00	1.00	.974	.980
fru-F-700157	VMP	MED	4	74	1.00	1.00	1.00	1.00	1.00
fru-F-800052	VMP	MED	10	91	1.00	1.00	1.00	1.00	1.00
fru-F-800083	VMP	MED	6	64	1.00	1.00	1.00	1.00	1.00
fru-F-900027	VMP	MED	8	67	.364	1.00	1.00	.788	.811
fru-F-900039	VMP	MED	10	71	1.00	1.00	1.00	1.00	1.00
Gad1-F-000709	AOTU	LOB	21	31	1.00	1.00	1.00	1.00	1.00
Gad1-F-000777	AOTU	LOB	15	44	1.00	.955	.846	1.00	.964
Gad1-F-200218	AOTU	MED	5	42	1.00	1.00	1.00	1.00	1.00
Gad1-F-200389	AOTU	LOB	8	16	1.00	1.00	1.00	1.00	1.00
Gad1-F-200780	AOTU	LOB	10	33	1.00	1.00	1.00	1.00	1.00
Gad1-F-300536	LH	AL	10	44	1.00	1.00	1.00	1.00	1.00
Gad1-F-400023	AOTU	MED	4	55	1.00	1.00	1.00	1.00	1.00
Gad1-F-500071	AOTU	LOB	4	11	0.00	0.00	0.00	0.00	0.00
Gad1-F-500088	LH	AL	13	42	1.00	1.00	1.00	1.00	1.00
Gad1-F-500568	LH	AL	18	44	1.00	1.00	1.00	1.00	1.00
Gad1-F-600331	AOTU	LOB	6	19	1.00	.950	.833	1.00	.960
Gad1-F-600560	CAL	AL	1	54	1.00	1.00	1.00	1.00	1.00
Gad1-F-600676	LH	AL	11	157	1.00	1.00	1.00	1.00	1.00
Gad1-F-700055	AOTU	MED	3	59	1.00	1.00	1.00	1.00	1.00
Gad1-F-800354	LH	AL	5	27	1.00	1.00	1.00	1.00	1.00
Gad1-F-800392	AOTU	LOB	5	32	1.00	.889	.200	1.00	.892
Gad1-F-900119	AOTU	LOB	9	20	1.00	1.00	1.00	1.00	1.00
Gad1-F-900515	LH	AL	13	70	1.00	1.00	1.00	1.00	1.00
TH-F-000048	PB	CVLP,ID FP,VMP	67	75	.805	1.00	1.00	.787	.887
Trh-F-300080	LH	AL	13	20	1.00	1.00	1.00	1.00	1.00
Trh-F-400067	LH	AL	9	13	1.00	1.00	1.00	1.00	1.00
Trh-F-500027	LH	AL	7	12	1.00	1.00	1.00	1.00	1.00
Trh-F-500049	LH	AL	12	16	1.00	1.00	1.00	1.00	1.00
Trh-F-500059	LH	AL	12	50	1.00	1.00	1.00	1.00	1.00
Trh-F-500077	LH	AL	4	20	1.00	1.00	1.00	1.00	1.00
Trh-F-600092	LH	AL	15	23	1.00	1.00	1.00	1.00	1.00
Trh-F-600104	LH	AL	12	15	1.00	1.00	1.00	1.00	1.00
Trh-F-700032	LH	AL	4	16	1.00	1.00	1.00	1.00	1.00
VGlut-F-000259	LH	AL	38	138	1.00	1.00	1.00	1.00	1.00
VGlut-F-000370	LH	AL	12	146	1.00	1.00	1.00	1.00	1.00

VGlut-F-300584	PB	CVLP,DMP	19	83	.667	.819	.105	.987	.814
VGlut-F-400245	CAL	AL	7	47	1.00	1.00	1.00	1.00	1.00
VGlut-F-400634	LH	AL	15	26	1.00	1.00	1.00	1.00	1.00
VGlut-F-500092	LH	AL	11	27	1.00	1.00	1.00	1.00	1.00
VGlut-F-500853	VLP	LOB	4	21	1.00	1.00	1.00	1.00	1.00
VGlut-F-600669	LH	AL	11	51	1.00	1.00	1.00	1.00	1.00
VGlut-F-600757	LH	AL	9	34	1.00	1.00	1.00	1.00	1.00
VGlut-F-700021	CAL	AL	2	35	1.00	1.00	1.00	1.00	1.00
VGlut-F-700072	CAL	AL	2	32	1.00	1.00	1.00	1.00	1.00
VGlut-F-700163	VLP	LOB	2	39	0.00	.917	0.00	.564	.537
VGlut-F-700230	VLP	LOB	8	27	.500	.806	.250	.926	.771
VGlut-F-700402	VLP	LOB	10	12	1.00	1.00	1.00	1.00	1.00
VGlut-F-800076	VLP	LOB	6	25	.667	1.00	1.00	.880	.903
VGlut-F-800224	VLP	LOB	11	67	.688	1.00	1.00	.917	.930
VGlut-F-800284	LH	AL	18	21	1.00	1.00	1.00	1.00	1.00
VGlut-F-800305	VLP	LOB	5	17	1.00	1.00	1.00	1.00	1.00

Tabel S2. Polarity Identification of Complex Neurons in the Dataset

Neuron ID	Brain Region		No. of Terminals		Precision		Recall		Accuracy
	Axon	Dendrite	Axon	Dendrite	Axon	Dendrite	Axon	Dendrite	
Cha-F-000014	IDFP	FB,PB	14	36	1.00	1.00	1.00	1.00	1.00
Cha-F-000023	FB,NO	PB	14	6	1.00	.556	.714	1.00	.789
Cha-F-000031	IDFP	FB,PB	16	37	1.00	.720	.125	1.00	.731
Cha-F-000050	PB	CCP,VMP	9	39	0.00	.804	0.00	.974	.787
Cha-F-000098	IDFP	FB,PB	9	24	1.00	1.00	1.00	1.00	1.00
Cha-F-000106	FB,NO	PB	23	11	1.00	.478	.478	1.00	.647
Cha-F-000112	FB,NO	PB	9	5	1.00	1.00	1.00	1.00	1.00
Cha-F-000423	EB,NO	PB	34	6	1.00	1.00	1.00	1.00	1.00
Cha-F-100032	IDFP	FB,PB	12	31	1.00	1.00	1.00	1.00	1.00
Cha-F-100041	IDFP	FB,PB	19	67	1.00	1.00	1.00	1.00	1.00
Cha-F-100065	IDFP	FB,PB	14	42	1.00	1.00	1.00	1.00	1.00
Cha-F-100117	PB	CCP,VMP	9	49	0.00	.833	0.00	.957	.804
Cha-F-100206	FB,NO	PB	18	14	1.00	.750	.765	1.00	.862
Cha-F-200009	IDFP	FB,PB	14	20	1.00	1.00	1.00	1.00	1.00
Cha-F-200013	IDFP	FB,PB	15	43	1.00	.759	.071	1.00	.764
Cha-F-200046	IDFP	FB,PB	8	18	1.00	1.00	1.00	1.00	1.00
Cha-F-200068	IDFP	FB,PB	15	48	1.00	.857	.467	1.00	.873
Cha-F-200084	IDFP	FB,PB	10	18	1.00	.810	.556	1.00	.846
Cha-F-300072	FB,NO	PB	14	13	1.00	1.00	1.00	1.00	1.00
Cha-F-300152	FB,NO	PB	13	7	1.00	.389	.154	1.00	.450
Cha-F-300160	IDFP	FB,PB	14	20	1.00	.519	.071	1.00	.536
Cha-F-400006	FB,NO	PB	19	8	1.00	.636	.789	1.00	.846
Cha-F-400012	IDFP	FB,PB	12	60	1.00	.866	.250	1.00	.871
Cha-F-400017	IDFP	FB,PB	11	50	1.00	1.00	1.00	1.00	1.00
Cha-F-400025	FB,NO	PB	14	10	1.00	1.00	1.00	1.00	1.00
Cha-F-400260	FB,NO	PB	17	11	1.00	1.00	1.00	1.00	1.00
Cha-F-500009	EB,NO	PB	20	10	1.00	1.00	1.00	1.00	1.00
Cha-F-500028	IDFP	FB,PB	21	41	1.00	.784	.421	1.00	.814
Cha-F-500046	IDFP,PB	EB	13	8	.429	0.00	.500	0.00	.300
Cha-F-500056	CAL,LH	AL	28	24	1.00	1.00	1.00	1.00	1.00
Cha-F-500109	IDFP	FB,PB	9	52	1.00	1.00	1.00	1.00	1.00
Cha-F-500285	FB,NO	PB	15	7	1.00	1.00	1.00	1.00	1.00
Cha-F-600001	IDFP	FB,PB	14	47	1.00	.836	.357	1.00	.850
Cha-F-700086	IDFP	FB,PB	10	32	1.00	1.00	1.00	1.00	1.00

fru-F-100063	EB,NO	PB	12	8	1.00	1.00	1.00	1.00	1.00
fru-F-400276	CAL,LH	AL	17	54	1.00	.964	.882	1.00	.972
fru-F-500176	CAL,LH	AL	13	38	1.00	1.00	1.00	1.00	1.00
fru-F-700239	CAL,LH	AL	5	11	1.00	1.00	1.00	1.00	1.00
fru-M-400292	CAL,LH	AL	16	40	1.00	1.00	1.00	1.00	1.00
fru-M-400387	CAL,LH	AL	12	11	1.00	1.00	1.00	1.00	1.00
Gad1-F-000056	FB,NO	PB	13	4	1.00	.500	.667	1.00	.750
Gad1-F-000066	FB,NO	PB	18	8	1.00	.381	.278	1.00	.500
Gad1-F-000157	FB,NO	PB	17	14	1.00	.800	.812	1.00	.893
Gad1-F-000167	CAL,LH	AL	14	20	1.00	1.00	1.00	1.00	1.00
Gad1-F-000172	FB,NO	PB	15	20	1.00	1.00	1.00	1.00	1.00
Gad1-F-000671	CAL,LH	AL	11	21	1.00	1.00	1.00	1.00	1.00
Gad1-F-100004	IDFP	FB,PB	8	23	1.00	1.00	1.00	1.00	1.00
Gad1-F-100134	CAL,LH	AL	9	16	1.00	1.00	1.00	1.00	1.00
Gad1-F-200375	EB,NO	PB	13	10	1.00	1.00	1.00	1.00	1.00
Gad1-F-300027	IDFP	FB,PB	6	22	1.00	.875	.500	1.00	.889
Gad1-F-300029	IDFP	FB,PB	9	19	1.00	.760	.143	1.00	.769
Gad1-F-300066	IDFP	FB,PB	19	29	1.00	.585	.056	1.00	.595
Gad1-F-300099	IDFP	FB,PB	3	23	1.00	1.00	1.00	1.00	1.00
Gad1-F-300121	FB,NO	PB	12	5	1.00	1.00	1.00	1.00	1.00
Gad1-F-300123	IDFP	FB,PB	17	35	1.00	1.00	1.00	1.00	1.00
Gad1-F-300189	FB,NO	PB	10	12	1.00	1.00	1.00	1.00	1.00
Gad1-F-300520	CAL,LH	AL	11	12	1.00	1.00	1.00	1.00	1.00
Gad1-F-400005	FB,NO	PB	11	7	1.00	.875	.909	1.00	.944
Gad1-F-400017	IDFP	FB,PB	7	26	1.00	1.00	1.00	1.00	1.00
Gad1-F-400104	FB,NO	PB	15	2	1.00	.286	.667	1.00	.706
Gad1-F-400312	FB,NO	PB	20	6	1.00	1.00	1.00	1.00	1.00
Gad1-F-400385	FB,NO	PB	17	11	1.00	1.00	1.00	1.00	1.00
Gad1-F-400400	PB	CCP,VMP	7	26	0.00	.731	0.00	.950	.704
Gad1-F-500035	IDFP	FB,PB	13	58	1.00	1.00	1.00	1.00	1.00
Gad1-F-500065	IDFP	FB,PB	8	35	1.00	.829	.125	1.00	.833
Gad1-F-500299	CAL,LH	AL	13	80	1.00	1.00	1.00	1.00	1.00
Gad1-F-500312	CAL,LH	AL	13	68	1.00	1.00	1.00	1.00	1.00
Gad1-F-500661	CAL,LH	AL	17	11	1.00	1.00	1.00	1.00	1.00
Gad1-F-600003	FB,NO	PB	5	6	.400	0.00	.800	0.00	.364
Gad1-F-600006	FB,NO	PB	8	4	1.00	1.00	1.00	1.00	1.00
Gad1-F-600025	EB,NO	PB	11	9	1.00	1.00	1.00	1.00	1.00

Gad1-F-600033	IDFP	FB,PB	13	26	1.00	.774	.417	1.00	.806
Gad1-F-600077	FB,NO	PB	7	4	1.00	1.00	1.00	1.00	1.00
Gad1-F-600081	IDFP	FB,PB	10	34	1.00	.850	.400	1.00	.864
Gad1-F-600084	IDFP,PB	EB	9	24	1.00	.786	.333	1.00	.806
Gad1-F-700120	CAL,LH	AL	35	20	1.00	1.00	1.00	1.00	1.00
Gad1-F-700125	FB,NO	PB	10	5	1.00	1.00	1.00	1.00	1.00
Gad1-F-700150	CAL,LH	AL	10	9	1.00	1.00	1.00	1.00	1.00
Gad1-F-700275	CAL,LH	AL	8	24	1.00	1.00	1.00	1.00	1.00
Gad1-F-800013	IDFP	FB,PB	18	55	1.00	1.00	1.00	1.00	1.00
Gad1-F-800025	IDFP	FB,PB	12	42	1.00	1.00	1.00	1.00	1.00
Gad1-F-800046	FB,NO	PB	20	5	1.00	1.00	1.00	1.00	1.00
Gad1-F-800113	FB,NO	PB	17	12	1.00	1.00	1.00	1.00	1.00
Gad1-F-800139	FB,NO	PB	19	5	1.00	1.00	1.00	1.00	1.00
Gad1-F-900011	IDFP	FB,PB	13	37	1.00	.949	.833	1.00	.959
Gad1-F-900035	CAL,LH	AL	11	9	1.00	1.00	1.00	1.00	1.00
npf-F-100003	CAL,LH	AL	12	21	1.00	1.00	1.00	1.00	1.00
npf-F-100004	CAL,LH	AL	14	34	1.00	1.00	1.00	1.00	1.00
npf-F-100009	CAL,LH	AL	12	18	1.00	1.00	1.00	1.00	1.00
npf-F-100010	CAL,LH	AL	16	15	1.00	1.00	1.00	1.00	1.00
npf-F-100011	CAL,LH	AL	17	21	1.00	1.00	1.00	1.00	1.00
npf-F-200001	CAL,LH	AL	13	11	1.00	1.00	1.00	1.00	1.00
npf-F-200003	CAL,LH	AL	20	17	1.00	1.00	1.00	1.00	1.00
npf-F-200008	CAL,LH	AL	13	16	1.00	1.00	1.00	1.00	1.00
npf-F-200018	CAL,LH	AL	17	28	1.00	1.00	1.00	1.00	1.00
npf-F-200042	CAL,LH	AL	12	16	1.00	1.00	1.00	1.00	1.00
npf-F-200044	CAL,LH	AL	15	11	1.00	1.00	1.00	1.00	1.00
npf-M-100010	CAL,LH	AL	13	18	1.00	1.00	1.00	1.00	1.00
Tdc2-F-200009	IDFP	FB,PB	4	25	1.00	1.00	1.00	1.00	1.00
Tdc2-F-300003	IDFP	FB,PB	8	29	1.00	1.00	1.00	1.00	1.00
Tdc2-F-300014	IDFP	FB,PB	5	34	1.00	1.00	1.00	1.00	1.00
Tdc2-F-300036	IDFP	FB,PB	5	30	1.00	1.00	1.00	1.00	1.00
Tdc2-F-300042	IDFP	FB,PB	3	34	1.00	1.00	1.00	1.00	1.00
Tdc2-F-400002	IDFP	FB,PB	3	22	1.00	1.00	1.00	1.00	1.00
Tdc2-F-400009	IDFP	FB,PB	2	32	1.00	1.00	1.00	1.00	1.00
Tdc2-F-400026	CAL,LH	AL,VMP	157	269	1.00	1.00	1.00	1.00	1.00
Tdc2-F-500000	IDFP	FB,PB	6	32	1.00	1.00	1.00	1.00	1.00
Tdc2-F-600000	IDFP	FB,PB	4	34	1.00	1.00	1.00	1.00	1.00

VGlut-F-000485	CAL,LH	AL	5	81	1.00	1.00	1.00	1.00	1.00
VGlut-F-200566	CAL,LH	AL	27	62	1.00	1.00	1.00	1.00	1.00
VGlut-F-200574	CAL,LH	AL	9	24	1.00	1.00	1.00	1.00	1.00
VGlut-F-300243	FB,NO	PB	9	3	1.00	1.00	1.00	1.00	1.00
VGlut-F-300517	FB,NO	PB	18	7	1.00	.353	.353	1.00	.522
VGlut-F-300596	CAL,LH	AL	18	10	1.00	1.00	1.00	1.00	1.00
VGlut-F-400664	CAL,LH	AL	13	9	1.00	1.00	1.00	1.00	1.00
VGlut-F-500626	CAL,LH	AL	9	7	1.00	1.00	1.00	1.00	1.00
VGlut-F-600146	CAL,LH	AL	8	18	1.00	1.00	1.00	1.00	1.00
VGlut-F-600243	CAL,LH	AL	14	5	1.00	1.00	1.00	1.00	1.00
VGlut-F-600248	CAL,LH	AL	13	19	1.00	1.00	1.00	1.00	1.00
VGlut-F-600751	CAL,LH	AL	6	13	1.00	1.00	1.00	1.00	1.00
VGlut-F-700285	CAL,LH	AL	7	15	1.00	1.00	1.00	1.00	1.00
VGlut-F-700494	CAL,LH	AL	14	8	1.00	1.00	1.00	1.00	1.00
VGlut-F-700547	CAL,LH	AL	16	28	1.00	1.00	1.00	1.00	1.00
VGlut-F-800097	CAL,LH	AL	7	10	1.00	1.00	1.00	1.00	1.00

Table S3. Additional 22 Test Neurons Not in the Dataset

Neuron ID	Brain Region		No. of Terminals		Precision		Recall		Accuracy
	Axon	Dendrite	Axon	Dendrite	Axon	Dendrite	Axon	Dendrite	
Gad1-F-800048	BU	AOTU	1	5	1.00	1.00	1.00	1.00	1.00
Cha-F-600054	BU	AOTU	1	3	0.500	1.00	1.00	0.667	0.750
Cha-F-200155	BU	AOTU	2	3	1.00	1.00	1.00	1.00	1.00
Cha-F-800034	BU	AOTU	2	2	1.00	1.00	1.00	1.00	1.00
Gad1-F-200129	BU	AOTU	2	5	1.00	1.00	1.00	1.00	1.00
Gad1-F-700209	BU	AOTU	1	13	1.00	1.00	1.00	1.00	1.00
Cha-F-000503	BU	AOTU	3	12	1.00	1.00	1.00	1.00	1.00
Gad1-F-400100	BU	AOTU	1	6	1.00	1.00	1.00	1.00	1.00
Gad1-F-100297	BU	AOTU	1	10	1.00	1.00	1.00	1.00	1.00
Gad1-F-000099	BU	AOTU	2	7	1.00	1.00	1.00	1.00	1.00
Gad1-F-000140	BU	AOTU	2	7	1.00	1.00	1.00	1.00	1.00
Cha-F-100098	BU	AOTU	3	4	1.00	1.00	1.00	1.00	1.00
Cha-F-100260	SIP, SLP	MB	17	30	1.00	1.00	1.00	1.00	1.00
VGlut-F-300494	VMP	MED	20	113	1.00	1.00	1.00	1.00	1.00
fru-F-400331	VMP	MED	32	78	1.00	1.00	1.00	1.00	1.00
VGlut-F-300446	VMP	MED	8	45	1.00	0.938	0.625	1.00	0.943
fru-F-300054	VMP	MED	12	81	1.00	1.00	1.00	1.00	1.00
fru-F-400249	VMP	MED	10	88	0.345	1.00	1.00	0.784	0.806
fru-F-400316	VMP	MED	11	52	1.00	1.00	1.00	1.00	1.00
fru-F-900040	VMP	MED	7	51	1.00	1.00	1.00	1.00	1.00
Cha-F-300436	AOTU	MED	4	49	1.00	1.00	1.00	1.00	1.00
Cha-F-700272	AOTU	MED	4	19	1.00	1.00	1.00	1.00	1.00
